# Hedgehog, Chamomile, and Multipetal Polymeric Structures on the Nanoparticle Surface: Computer Modelling

**DOI:** 10.3390/molecules27238535

**Published:** 2022-12-04

**Authors:** Zakhar R. Saraev, Alexei A. Lazutin, Valentina V. Vasilevskaya

**Affiliations:** 1 A.N. Nesmeyanov Institute of Organoelement Compounds, Russian Academy of Sciences, Vavilova St. 28, Moscow 119991, Russia; 2 Moscow Institute of Physics and Technology, National Research University, Institutskiy per. 9, Dolgoprudny 141701, Russia; 3 Chemistry Department, M. V. Lomonosov Moscow State University, Leninskie Gory, Moscow 119991, Russia

**Keywords:** amphiphilic homopolymers, self-assembly, nanoparticles

## Abstract

A single spherical nanoparticle coated with a densely grafted layer of an amphiphilic homopolymer with identical A-graft-B monomer units was studied by means of coarse-grained molecular dynamics. In solvent, selectively poor for mainchain and good for pendant groups; the grafted macromolecules self-assemble into different structures to form a complex pattern on the nanoparticle surface. We distinguish hedgehog, multipetalar, chamomile, and densely structured shells and outline the area of their stability using visual analysis and calculate aggregation numbers and specially introduced order parameters, including the branching coefficient and relative orientation of monomer units. For the first time, the branching effect of splitting aggregates along with the distance to the grafting surface and preferred orientation of the monomer units with rearrangements of the dense compacted shell was described. The results explain the experimental data, are consistent with the analytical theory, and are the basis for the design of stimulus-sensitive matrix-free composite materials.

## 1. Introduction

Nanoparticles, coated with a polymer layer, are included in applications in a wide range of the foremost areas of research, including drug delivery [1,2,3,4,5,6,7,8], the oil industry [9,10,11,12], and nanocomposite production [13,14,15,16]. Polymer-coated nanoparticles, being protected from aggregation, serve as effective fillers [17,18,19], and have recently been used to design so-called matrix-free composite materials (MFCMs) [20,21]. MFCMs lack a polymer matrix and consist exclusively of grafted polymer nanoparticles. Alternative names of such materials are one component polymer-grafted nanoparticles [22] or pure polymer-grafted nanoparticle constructs [23].

The local structure of MFCMs, and hence the properties, are determined not only by the composition (nanoparticle and polymer), but also by the structure of the grafted layer, which controls the processes of nanoparticle self-assembly. In this sense, MFCMs can be safely considered as a typical example of so-called bottom-up construction [24,25,26].

The bottom-up approach involves the creation of heterogeneous materials arranged in periodic and/or hierarchical structures through the design of their smallest components. The structure-controlling parameters are the size and shape of nanoparticles; their functionalities, mechanism, and orientation are dependent on mutual interactions [27]. The orientation dependence of the nanoparticle interactions manifests itself in cases of nanoparticles with a heterogeneous surface and leads to crucial changes in local self-assembly. Spherical nanoparticles with a homogeneous surface self-assemble into tightly packed structures with regular, such as face-centered cubic and hexagonal, lattices [28,29]. Spherical nanoparticles, with a Janus-like surface, form micelles, cylinders, and lamellae [30]. More diverse structures can be found in a solution of nanoparticles, which have a non-spherical shape and/or complex patchy or patterned surface [31,32,33]. 

Grafting a polymer onto the nanoparticle surface allows us not only to create certain patterns, but also to rearrange them under the influence of external factors [34]. The restructuring of the nanoparticle pattern undoubtedly causes the restructuring of the bottom-up structure assembled by such nanoparticles [34,35]. 

The simplest case is nanoparticles coated with a flexible homopolymer layer. In a good solvent, the grafted polymers create a protective shell of swollen macromolecules; these hairy nanoparticles, as opposed to the ordinary spherical ones mentioned above, are able form regular lattices [36,37]. In a poor solvent, the polymer chains collapse and, depending on the graft density and the macromolecular length, form either a homogeneous shell or various heterogeneous patterns [38,39]. Accordingly, the nanoparticles coated with a collapsed polymer will be assemble into either regular structures or complex heterogeneous aggregates [40,41]. 

More diverge morphologies arise when nanoparticles are coated with different macromolecules and/or macromolecules with a complex composition and architecture [42,43,44].

Cryo-electron tomography visualization of polystyrene particles coated by polyanionic chains in the presence of polycation-b-PEO copolymers [45,46,47] reveals the cylindrical strands and thin lamellae diverging from the nanoparticle surface. These structures are the result of interpolyelectrolyte complexation and form effective comb-like macromolecules with a hydrophobic backbone and hydrophilic PEO sidechains.

Diverging cylindrical filaments and lamellae on the surface of spherical nanoparticles have been observed when modeling nanoparticles coated with an amphiphilic homopolymer [48,49]. Amphiphilic homopolymers are macromolecules with complex monomer units containing groups with different affinities to the solvent [50,51,52,53,54,55]. Such macromolecules are also called amphiphilic at the level of single monomer units or macromolecules with local amphiphilicity [50]. Polymer surfactants and some comb-like macromolecules can also be classified as amphiphilic homopolymers [52]. Even though amphiphilic homopolymers include a variety of macromolecules, with amphiphilicity due to different interactions and natures, the characteristic features of their behavior can be described in the framework of a simple A-graft-B model [50,53,54,55].

In this context, spherical nanoparticles coated with an amphiphilic homopolymer [48,49,56] can serve as a coarse-grained model to determine the conditions for the formation of certain structures and to identify areas of stability. In a computer experiment, we observed both divergent cylindrical filaments and lamellae and named them a hedgehog and multipetaler, respectively. The structures are formed thanks to the complex A-graft-B monomer units and were found in the solvents with different qualities and selectivity to different groups of monomer units. The computer modeling of MFNC, made from coated nanoparticles, revealed that multipetalers join onto gel-like aggregates with nanoparticles as cross-links; on the other hand, hedgehogs remain separate even in solution with a very high concentration. Accordingly, the multipetaler solution exhibits a significant elastic response, while a solution of hedgehogs is a non-viscous liquid. Such a significant difference in the resulting structures is due to the difference in the number of open hydrophobic groups in the self-assembled patterns. The number of lamellae is very significant, which is much less in hedgehogs. 

Recent theoretical calculations have shown that under some conditions, the rearrangement between hedgehog and multipetal structures is possible with no crucial change in the quality of the solvent or its selectivity [56]. This gives broad prospects for precise design of MFNCs and for determining the conditions for their rapid and effective reconstruction.

The aim of this study was to model the spherical nanoparticles coated with an amphiphilic homopolymer and dissolved in a solvent that is selectively poor for main chains and good for pendant groups, to study the morphology of the grafted layers, to distinguish the most characteristic structures, and to reveal the areas of their stability on the state diagram. 

The paper is organized as follows. First, the model used in the computer experiment is described in Section 2. Next, we report the results of computer experiments on the molecular dynamics, on the basis of which a diagram of states was constructed, and the processes and possible causes of the restructuring of some structures into others in the evolution of the system were investigated (Section 3). Finally, a conclusion is provided in Section 4.

## 2. Model and Experimental Technique

**Figure 1:**
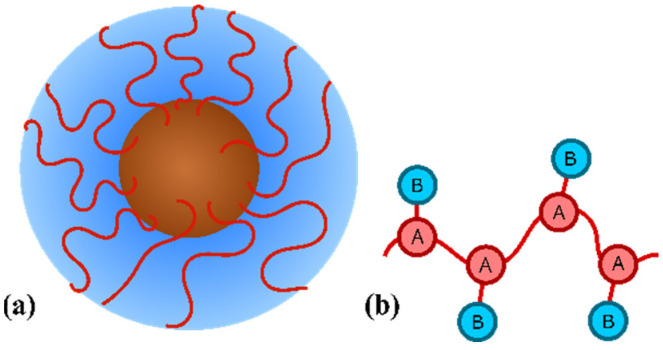
Model of a nanoparticle (**a**) coated with an amphiphilic homopolymer (**b**).

Amphiphilic homopolymers brushed on the spherical nanoparticle is within the framework of the coarse-grained model. A nanoparticle is represented as a hard sphere of radius *R*. *M* macromolecules having a degree of polymerization of *N* are grafted onto its surface (Figure 1a). In accordance with the aim of the article, these macromolecules are amphiphilic homopolymers, which are described using the “dumbbell” model [50,53,54,55].

In this model (Figure 1b), each monomeric unit consists of two beads, A and B. Hydrophobic A beads are connected to each other forming the backbone of the chain. Hydrophilic B beads are pendants attached to the backbone. Each chain is grafted onto the particle surface by fixing the A bead of its first monomeric unit to the surface. To distribute grafting points uniformly, they are placed at the nodes of the Fibonacci lattice (described in detail in [56]). The nanoparticle is immersed into a solvent, which is athermal for the nanoparticle and selective for the grafted amphiphilic homopolymer: it is athermal for B pendants and poor for the A main chain.

Molecular dynamics simulations were carried out using LAMMPS program package [57]. The Langevin thermostat, including an uncorrelated random force and a frictional force, was used to reproduce the canonical ensemble. The functional form of the force field includes terms that provide the excluded volume of the beads, ensure impenetrability of the nanoparticle surface, preserve the bond lengths, and consider solvent quality and selectivity.

The excluded volume of the beads was represented by the Weeks-Chandler-Andersen (WCA) potential (Equation (1)) [58]:

(1) $$U_{\mathrm{WCA}}\left(r_{ij}\right)=\{\begin{array}{cc} 4\varepsilon \left[{\left(\frac{\sigma }{r_{ij}}\right)}^{12}-{\left(\frac{\sigma }{r_{ij}}\right)}^{6}+\frac{1}{4}\right], & r_{ij}⩽r_{c}\\ 0, & r_{ij}{>r}_{c} \end{array},$$

where *r_ij_* is the distance between *i*-th and *j*-th beads (i,j=1,…,2MN), and $r_{c}=\sigma \sqrt[{6}]{2}$ is the cut-off distance. In this paper, we use a dimensionless measurement system commonly used in molecular simulations [59,60]. Parameters *σ* and *ε* of WCA potential and bead mass *m* were chosen as units of length, energy, and mass, accordingly; thus, by definition, $\sigma \overset{\mathrm{def}}{=}1,\epsilon \overset{\mathrm{def}}{=}1,m\overset{\mathrm{def}}{=}1$. All simulations were performed at a reduced temperature of unity: $k_{B}T=1$. 

Impenetrability of the nanoparticle surface was modeled by the shifted WCA potential (Equation (2)) [61]:

(2) $$U_{\mathrm{NP}}\left(r_{i}\right)=\{\begin{array}{cc} 4\varepsilon \left[{\left(\frac{\sigma }{\Delta_{i}+\frac{\sigma }{2}}\right)}^{12}-{\left(\frac{\sigma }{\Delta_{i}+\frac{\sigma }{2}}\right)}^{6}+\frac{1}{4}\right], & \Delta_{i}⩽r_{c}-\frac{\sigma }{2}\\ 0, & \Delta_{i}{>r}_{c}-\frac{\sigma }{2} \end{array},$$

where Δ*_i_* is the distance from the center of *i*-th bead (i, j=1,…,2MN) to the surface of the particle. 

The bonds between the main chain A beads and between A and B beads comprising the monomeric unit were constrained using the finite extensible nonlinear elastic (FENE) potential (Equation (3)) [62]:

(3) $$U_{\mathrm{bond}}\left(r_{ij}\right)=-\frac{K}{2}R_{b}^{2}\ln[1-{\left(\frac{r_{ij}}{R_{b}}\right)}^{2}],$$

where *r_ij_* is the length of the bond between *i*-th and *j*-th beads, *K* is a spring constant, and *R_b_* is the maximal bond length. Widely used values of these parameters, which are equal to *K* = 30 and *R_b_* = 1.5, were applied to keep bond length values in unity ($\langle r_{ij}\rangle =0.97$) [63]. 

Polymer-solvent interactions were implicitly considered by the effective Yukawa-type potential acting between all the beads (Equation (4)):

(4) $$U_{\alpha \beta }\left(r_{ij}\right)=\{\begin{array}{cc} \varepsilon_{\alpha \beta }\left(\frac{e^{-{\kappa r}_{c}}}{r_{ij}}-\frac{e^{-{\kappa r}_{y}}}{r_{y}}\right), & r_{ij}⩽r_{y}\\ 0, & r_{ij}{>r}_{y} \end{array}\},$$

where *r_ij_* is the distance between *i*-th and *j*-th beads, κ = 1.2 is the inverse screening length, *r_y_* = 4 is the cut-off distance, *α* and *β* are types of *i*-th and *j*-th beads, accordingly (α, β=A, B), and *ε_αβ_* are the energy parameters of interaction between beads of *α* and *β* types. The energy parameters can be positive, describing a mutual repulsion between the beads, and negative, describing their attraction. The values of these parameters were chosen in accordance with the aim of the article. Since the solvent is athermal for B beads, the only interaction between them is the excluded volume interaction, and the parameter for the Yukawa-type potential was set to zero (*ε_BB_* = 0). Hydrophobic A beads effectively attract each other; thus, their interaction parameter is negative (*ε_AA_* < 0). Hydrophilic and hydrophobic beads tend to separate from each other, and a positive interaction parameter (*ε_AB_* > 0) ensures this behavior.

Simulations were performed for the nanoparticle with a radius *R* = 5 grafted with *M* = 100 amphiphilic chains with a polymerization degree *N* = 50 and placed into a cubic simulation box with the edge of L = 150 and periodic boundary conditions. Energy parameters of interaction between A beads *ε_AA_* and between A and B beads *ε_AB_* were varied across a wide range $-10⩽\varepsilon_{BB}⩽0;\;0⩽\varepsilon_{AB}⩽10$ using a simulated annealing technique. Simulation starts with every polymer chain stretched out in the normal direction to the surface of the particle at the grafting point. Simulation was carried out without polymer-solvent interaction ($\varepsilon_{AB}{=\varepsilon }_{AA}{=\varepsilon }_{BB}=0$) until the drift of the average gyration radius of the chain disappears. Then, repulsion between A and B beads was increased in increments of ${\Delta \varepsilon }_{AB}=0.25$ until the final value $\varepsilon_{AB}=10$ was reached. Starting with the configuration obtained at each value of *ε_AB_*, attraction between A beads was increased by lowering the interaction parameter value by decrements of ${\Delta \varepsilon }_{AA}=-0.25$ until the final value $\varepsilon_{AA}=-10$ was reached. In this way, configurations for each pair of parameters values $\varepsilon_{AA}=0,-0.25,\ldots ,-10$ and $\varepsilon_{AB}=0,\;0.25,\ldots ,\;10$ were obtained. At each step, the simulation was performed for 5000τ, where $\tau =\sqrt{m\frac{\sigma^{2}}{\varepsilon }}$ with integration step 0.005τ. A time interval of least 2500τ was used to average the observed values. 

The results obtained are presented in the next section.

## 3. Results of Computer Modeling

### 3.1. Visualization

**Figure 2:**
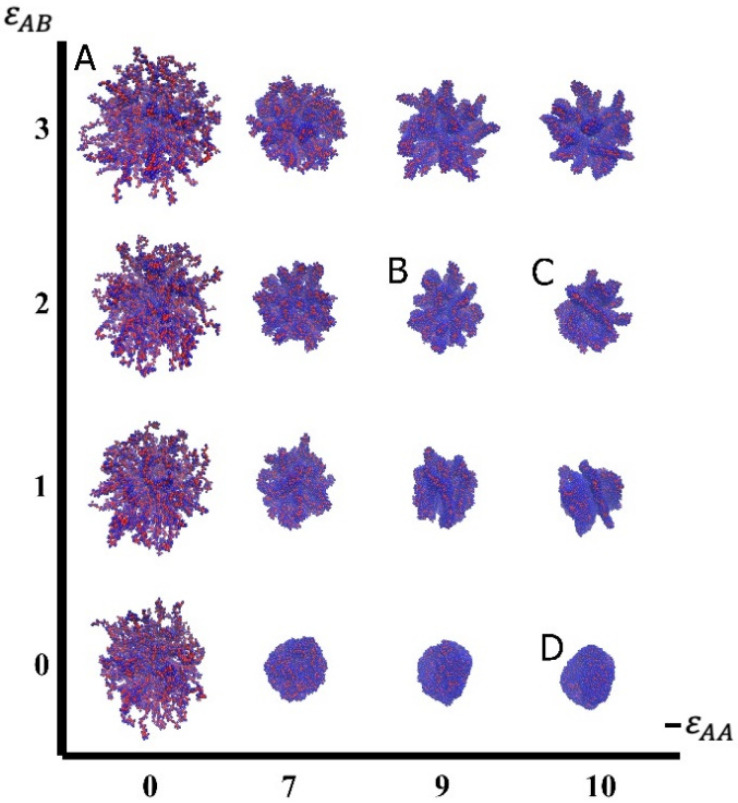
Instant snapshots presented as a diagram of $\varepsilon_{AA}$&$\varepsilon_{AB}$ variables. The color indicates the bead type: A beads are colored red and B beads are blue. Visually identified structures: swollen brush (**A**); hedgehog (**B**); multipetaler (**C**); compacted structured shell (**D**).

In Figure 2, the visual results are presented in the form of a morphological diagram with the variables attractive $\varepsilon_{AA}$ energy (effective solvent quality) vs. repulsive ε_AB_ interaction (effective surface activity). In the visual diagram, instantaneous images are displayed at certain fixed values of the energetical parameters $\varepsilon_{AA}$ and $\varepsilon_{AB}$. 

When inspecting the instant snapshots obtained in computer experiments, four different structures were identified that can form on the surface of a spherical nanoparticle. The first of them is a swollen brush (Figure 2A), with all the macromolecules in a coil state. This state is observed where the A-A attractive energy is small, and due to excluded volume interaction and A-B repulsion, macromolecules locally swell and stretch radially from the nanoparticle surface. The second structure is referred to as hedgehog (Figure 2B). In hedgehog, the neighboring macromolecules join into aggregates. The aggregates (“spikes”) consist of several macromolecules, have an elongated shape, and a core-shell structure. The inner parts are made of hydrophobic A beads of the main chain and hydrophilic B beads of pendants are placed on the outside and create dense protective shells. The number of macromolecules in the spike and, accordingly, the total number of spikes changes with A-A attraction and A-B repulsion. In the next distinguished structure (Figure 2C), the macromolecules self-assemble into thin membrane-like aggregates with hydrophobic A beads hidden inside and hydrophilic B beads located along the surface. These aggregates look like petals, and the structure itself has been called multipetaler. The fourth highlighted structure is the compacted structured shell (Figure 2D). In such structures, the macromolecules are assembled into a single cluster with a nanoparticle in the middle. The dense shells can be homogeneous or highly structured; they either have the shape of a spherical layer or form a complex relief. 

Considering visualization of the evolution of the system with changing energy parameters, the following can be concluded. The coiled brushes, observed at $\varepsilon_{AA}=0$, transform into structured states along with growth of A-A attractive interactions. In the case of the only excluded volume interactions between A and B ($\varepsilon_{AB}=0$), the coiled brush transforms to compacted structured shells; at strong A-B repulsion ($\varepsilon_{AB}=3$), the coiled brush adopts hedgehog structures. With intermediate values of $\varepsilon_{AB}$ ($\varepsilon_{AB}=1.2$), it is possible to distinguish hedgehogs (at weaker A-A attraction) and multipetalers (with stronger A-A attraction).

**Figure 3:**
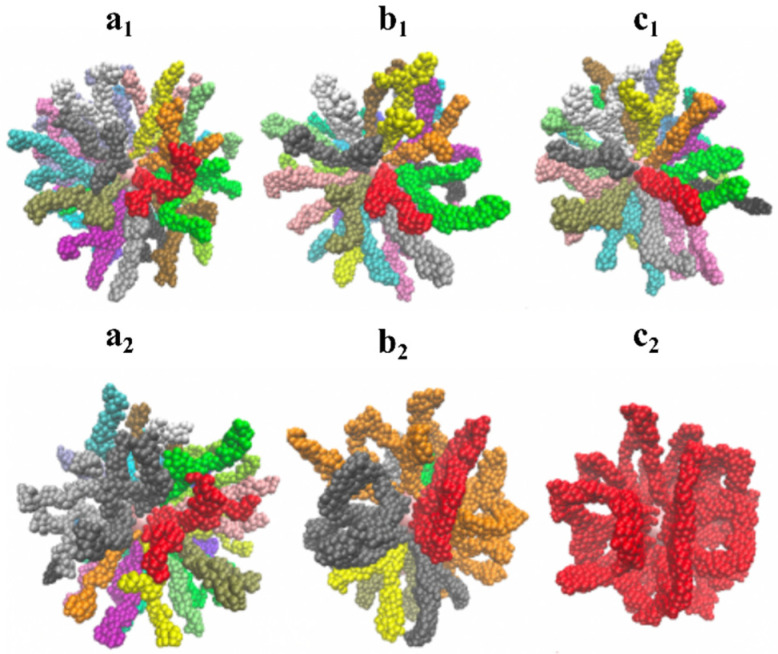
Instant snapshots of the obtained conformations, where the color indicates different clusters. Only A beads are shown. Top row: $\varepsilon_{AB}=3$ and $\varepsilon_{AA}=-8$ (**a_1_**), $\varepsilon_{AA}=-9$ (**b_1_**), $\varepsilon_{AA}=-10$ (**c_1_**); Bottom row: $\varepsilon_{AB}=1.5$ and $\varepsilon_{AA}=-6.5$ (**a_2_**), $\varepsilon_{AA}=-8$ (**b_2_**), $\varepsilon_{AA}=-10$ (**c_2_**).

The transition between different states proceeds smoothly through the formation of intermediate states. As seen in Figure 3, A beads are colored in accordance with belonging to a particular cluster, defined as a set of chains, in which at least two A beads of different macromolecules are located at a distance of less than 1.05 σ. 

Figure 3a_1_–c_1_ (strong A-B repulsion with $\varepsilon_{AB}$ = 3) depicts the transition states between different hedgehogs, i.e., structures with all macromolecules collected into prolonged clusters. It is seen that the clusters are not stick rods; they are flexible and wriggle in different directions. The total number of clusters decreases with the growth of A-A attractive interactions. Some clusters are split at the ends; others look as if they grow from the single root. 

In Figure 3a_2_–c_2_ ($\varepsilon_{AB}$ = 1.5), the sequence of transitions from hedgehog to multipetaler can be observed. With $\varepsilon_{AB}=-6.5$, all macromolecules are collected into prolonged clusters, which split at the ends. 

With $\varepsilon_{AB}$ = 1.5 and $\varepsilon_{AA}=-8$ (Figure 3b_2_), the total number of clusters is smaller. The adjacent aggregates bend and their ends are fused and form loops. The grafted layer becomes denser. Near the grafting surface, individual filaments converge so closely that, within the framework of the proposed computational method of cluster distribution, they belong to the same aggregate.

At $\varepsilon_{AB}$ = 1.5 and $\varepsilon_{AA}=-10$ (Figure 3c_2_), all macromolecules can be attributed to a single cluster. Some of the macromolecular filaments are connected as loops while others are fused into lamellae.

**Figure 4:**
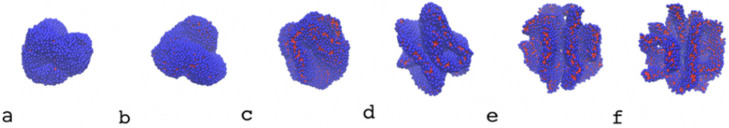
Sequence of transformation of a one-cluster structure along with an increase of $\varepsilon_{AB}$ = 0.0 (**a**), $\varepsilon_{AB}$ = 0.25 (**b**), $\varepsilon_{AB}$ = 0.5 (**c**),$\;\;\varepsilon_{AB}=$ 0.75 (**d**), $\varepsilon_{AB}=$ 1.5 (**e**), $\varepsilon_{AB}$ = 2.0 (**f**). $\varepsilon_{AA}=-10$.

The sequence of transformation of the compacted structured shell into a multipetaler structure at fixed strong $\varepsilon_{AA}=-10$ attraction and different $\varepsilon_{AB}$ values is presented in Figure 4. It is worthwhile to note that in all the pictured structures, the macromolecules are assembled into a single cluster, according to the procedure for determining the cluster described above.

When $\varepsilon_{AB}$= 0, the compacted shell is self-assembled in such a way that only hydrophilic B groups are found in the outer layer. The shell is quite embossed and convex parts can be distinguished. At $\varepsilon_{AB}$= 0.25 and 0.5, the outer layer of the shell becomes less uniform, and red regions of hydrophobic groups appear on the surface. Then ($\varepsilon_{AB}=$ 0.75), the shell splits into two parts, which we have called petals. A further growth in A-B repulsion leads to an increase in the total number of petals and their thinning. 

### 3.2. Quantitative Characteristics

To quantify the borderlines between the characteristic structural regions, we have calculated aggregation numbers A and their average values 〈A〉, introduced a branching coefficient β, and assessed the relative orientation *θ* of the A-B bonds.

These values allowed us to outline the beginning of chain aggregation and their completion, the transformation of individual components, and the structure as a whole.

The aggregation number A is the total number of macromolecules in one cluster. The average aggregation number 〈A〉 displays the average number of chains in all formed clusters at each moment of time and, thus, makes it possible to judge the state of the entire grafted layer. For instance, if an average aggregation number 〈A〉 is equal to 1, all chains are separated and do not create any cluster. In contrast, if the value 〈A〉 is equal to the total number M of polymeric chains grafted onto the nanoparticle 〈A〉≈ M, there is one cluster with the maximum possible aggregation number M, thus all chains are united.

The branching coefficient β is a slope coefficient on the dependence of branching ratio Rb on the distance from nanoparticle surface *L*. The branching ratio was set as Rb = $\frac{C_{l}\left(L\right)}{C_{l}\left(0\right)}$, where $C_{l}\left(0\right)$ is total number of clusters formed by macromolecules and $C_{l}\left(L\right)$ is the number of clusters formed by chains outside the spherical layers with width *L*. 

**Figure 5:**
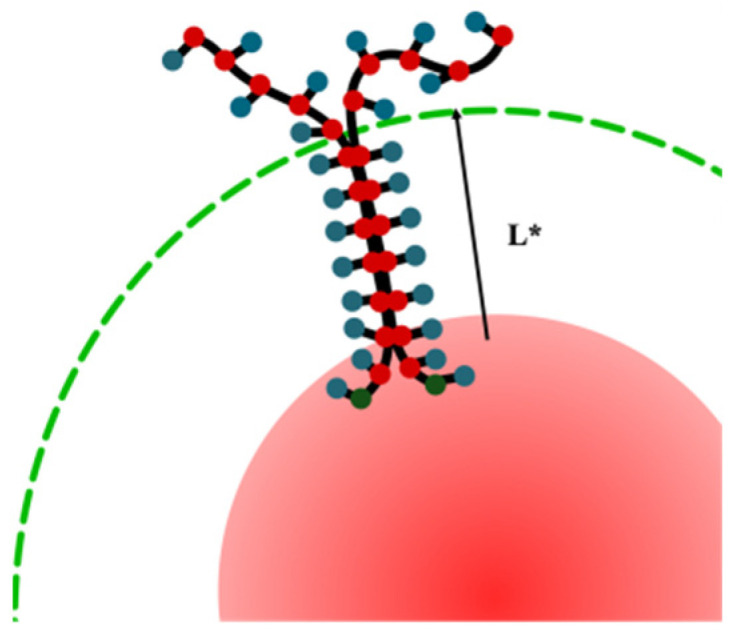
Scheme for calculating the branching ratio Rb. The parameter $C_{l}\left(0\right)$ is determined along the entire length of the macromolecules and in the depicted case it is equal to 1. The parameter $C_{l}\;\left(L\right)$ is determined by the areas outside the sphere *L* and in the depicted case it is equal to 1 for *L* < L* and 2 for *L* > L*.

The scheme of branching ratio Rb calculation is illustrated in Figure 5.

The *Rb* calculation is performed because the aggregation of the chains does not occur immediately along the entire length. It proceeds sequentially due to the radial expansion of the chains and a decrease in the polymer concentration along with the distance from the nanoparticle surface. The aggregation of macromolecules begins in the layer closest to the nanoparticle surface and, as the attraction increases, spreads into distant layers.

When calculating number of aggregates outside the spherical layer with width *L*, one can catch the branching effect and determine whether it is observed and at what distance from the nanoparticle it begins and ends. In addition, the branching ratio *Rb* and coefficient *β* allow us to indirectly determine the thickness of the grafted layer and evaluate its change during computer experiments.

The branching effect causes both an increase of the number of aggregates and a decrease in their average aggregation number (Figure 5). Quantitatively, the latter was characterized by calculating the average aggregation number ${\langle A\rangle }_{L}$ in the region outside the near-surface layer with a width *L*. With this definition: $\langle A\rangle ={\langle A\rangle }_{L=0}.$

**Figure 6:**
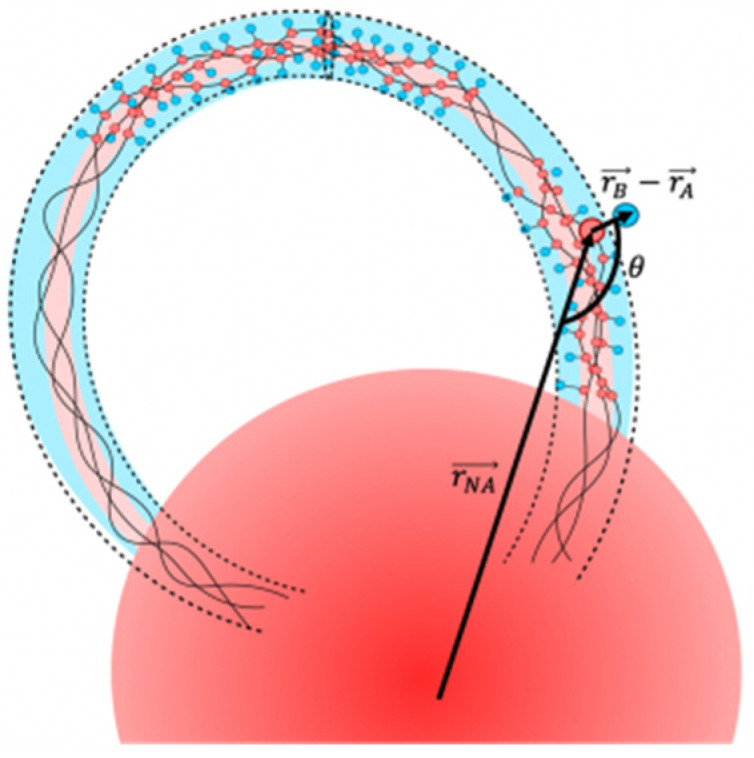
Schematic presentation of relative orientation of the A-graft-B monomer units ${\vec{r}}_{B}-{\vec{r}}_{A}$ with respect to the radius vector ${\vec{r}}_{NA}$. *θ* is an angle between these vectors.

Relative orientation *η* of the A-graft-B monomer unit with respect to the normal nanoparticle surface was calculated as follows (Equation (5)):

(5) $$\eta =\left|cos\left(\theta \right)\right|=\frac{\left|\left({\vec{r}}_{A}-{\vec{r}}_{B}\right)\cdot {\vec{r}}_{NA}\right|}{\left|{\vec{r}}_{A}-{\vec{r}}_{B}\right|\left|{\vec{r}}_{NA}\right|},$$

where ${\vec{r}}_{A}$ and ${\vec{r}}_{B}$ are radius-vectors of A and B beads of the monomer unit, respectively, and ${\vec{r}}_{NA}$ is radius vector connecting the centers of the A bead and the nanoparticle (see Figure 6). 

The relative orientation η is equal to zero when vector ${\vec{r}}_{AB}={\vec{r}}_{B}-{\vec{r}}_{A}$, which connects the A and B beads of the monomer unit, is perpendicular to the radius vector ${\vec{r}}_{NA}$ (*θ* = 90°); and η = 1 for the same and opposite directions of ${\vec{r}}_{AB}$ and ${\vec{r}}_{NA}$: *θ* = 0° or *θ* = 180°. The relative orientation η and its distribution over all monomeric units allows us to distinguish the preferred orientation of the surface-active A-graft-B monomer units and thereby quantify the boundaries between states with preferably different orientations. For example, in hedgehog spikes, the overwhelming number of monomer units have a relative orientation η close to zero: η~0; in the loops, the relative orientation η of monomer units varies across a wide interval from η~0 to η~1; in a dense shell with layered monomer units, for a significant part of monomer units the relative orientation η is about unity: η~1. 

### 3.3. Results of Calculations

**Figure 7:**
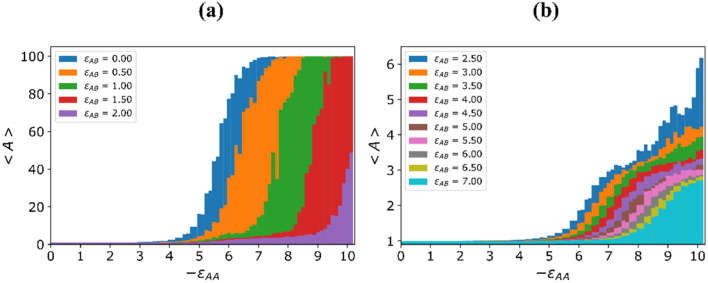
Dependency of the average aggregation number 〈A〉 on energy $\varepsilon_{AA}$ at different values of energy $\varepsilon_{AB}$: $\varepsilon_{AB}\leq 2$ (**a**), $\varepsilon_{AB}>2$ (**b**) The $\varepsilon_{AB}$ values are indicated in the inserts.

Figure 7 shows the dependencies of the average aggregation number 〈A〉 on the energy $\varepsilon_{AA}$ of A-A attraction at a fixed repulsion energy $\varepsilon_{AB}$ between A and B beads. 

The curves are divided into two groups. The first is when the aggregation process ends with the formation of a single cluster (Figure 7a) and the second is when clusters with a certain value of the aggregation number are observed at all $\varepsilon_{AA}$, i.e., much less than the total number of grafted macromolecules: 〈A〉≪M, (Figure 7b).

In all cases with small $\varepsilon_{AA}$ values, the aggregation number 〈A〉 is equal to unity: 〈A〉= 1. The attractive A-A interaction causes the aggregation of macromolecules, beginning with some values of $\varepsilon_{AA}^{C}$, the aggregation number 〈A〉 starts to increase. It is seen that the $\left|\varepsilon_{AA}^{C}\right|$ value increases along with $\varepsilon_{AB}$.

With $\varepsilon_{AB}\leq$ 0.5, 〈A〉 is a monotonous function of $\varepsilon_{AA}$, going to the maximum possible values of ${\langle A\rangle }_{max}=100$ within a narrow region. With $1\leq \varepsilon_{AB}\leq$1.5, the aggregation number 〈A〉 starts growth and reaches ${\langle A\rangle }_{max}=100$ at larger $\left|\varepsilon_{AA}^{C}\right|$. Here, one can distinguish the plateau region of an intermediate stable state with slightly increasing average aggregation numbers 〈A〉. At $\varepsilon_{AB}$ = 2, the plateau region becomes wider and the maximum value ${\langle A\rangle }_{max}$ is two-fold smaller and is reached at bigger $\left|\varepsilon_{AA}\right|$. 

At larger $\varepsilon_{AB}$ (Figure 7b), the maximum aggregation number ${\langle A\rangle }_{max}$ varies within a narrow interval: from ${\langle A\rangle }_{max}$~6 at $\varepsilon_{AB}$ = 2.5 till ${\langle A\rangle }_{max}$~3 at $\varepsilon_{AB}$ = 7. The plateau of the intermediate stable state shrinks along with growth of $\varepsilon_{AB}$, and for $\varepsilon_{AB}$ ≥ 3, it disappears.

**Figure 8:**
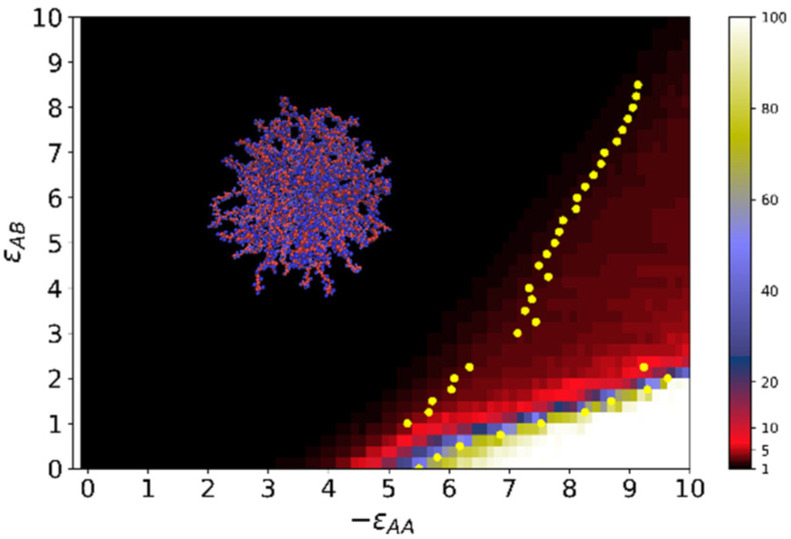
A color map of the average aggregation number 〈A〉. The color scale is indicated on the right.

The results are summarized as a color map of $\varepsilon_{AB}$&$\varepsilon_{AA}$ variables, where aggregation numbers 〈A〉 are marked by different colors in accordance with the color scale (Figure 8). 

Most of the color map is a black area with 〈A〉 = 1. It is the area of the swollen brush. The yellow dots are the inflection points on the dependencies of 〈A〉 on energy $\varepsilon_{AA}$, which were calculated numerically. The area between the two dotted yellow curves, where the aggregation number 〈A〉 changes from 2 up to 20, includes the hedgehog and transition regions. In the transition region, the hedgehog is rearranged, including the formation of loops. It can be treated as chamomile. The area where all macromolecules are collected in one cluster is highlighted in white. There can be both compacted structured shells and multipetalers when macromolecules are assembled into thin membranes that diverge from the surface.

**Figure 9:**
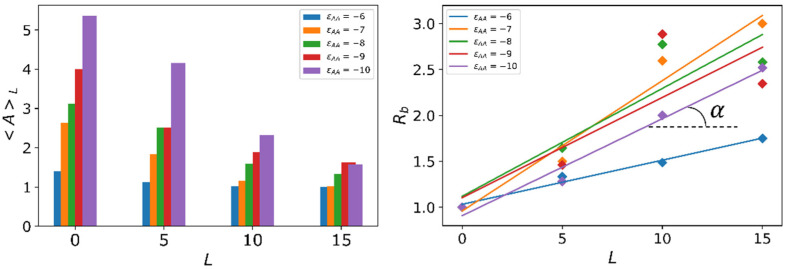
Dependency of the average aggregation number ${\langle A\rangle }_{L}$ and branching ratio Rb as a function of the distance *L* from the nanoparticle surface. $\varepsilon_{AB}=3$. β=tgα is a slope of a linear fit of the latter.

Figure 9 shows the aggregation number ${\langle A\rangle }_{L}$ and branching ratio Rb as functions of distance *L* from the nanoparticle surface. In all the cases, the aggregation number ${\langle A\rangle }_{L}$ is maximal right on the nanoparticle surface: *L* = 0. The average aggregation number ${\langle A\rangle }_{L}$ decreases and branching ratio Rb increases with an increase in distance *L* from the nanoparticle surface.

**Figure 10:**
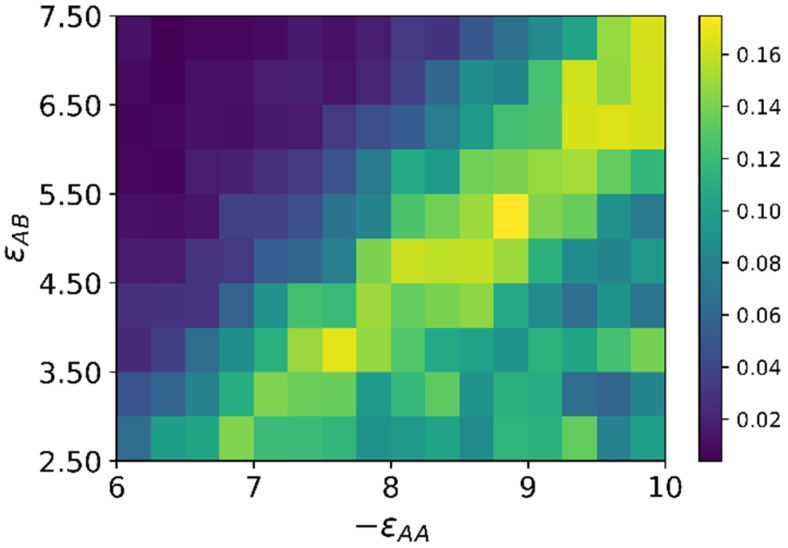
Color map of branching coefficient *β*. The color scale is indicated on the right.

The minimum increase in the number of aggregates is observed at $\varepsilon_{AA}$ = −6, the most significant increase is almost three times at $\varepsilon_{AA}$ = −7. The quantitative characteristic of this growth (the slope *β* of the linearly approximated dependence of Rb on *L*) is presented in the form of a color map in Figure 10.

The branching coefficient *β* is equal to zero only in the upper part of diagram above a certain diagonal from the lower left corner to the upper right, i.e., when the attractive energy between A beads is not enough to overcome the conformation entropy of macromolecules and repulsion of the A and B groups, and force macromolecules to join into clusters. In this case, the aggregation number ${\langle A\rangle }_{L}$ is equal to unity directly at the surface of the nanoparticle and remains far away from it up to *L* = 15. 

In all other cases, as soon as the macromolecules begin to combine into aggregates, the aggregation number decreases with increasing distance from the surface. This is not surprising; with increasing *L*, the relative grafting density decreases, and chain aggregation requires an ever-greater attractive A-A energy. The area with the maximum value of the branching coefficient *β*, i.e., with the most significant increase in the number of aggregates at *L* = 15 compared with *L* = 0, is also a diagonal line (yellow cells in the color map in Figure 10). Thus, an increase in the parameter $\left|\varepsilon_{AA}\right|$ at a fixed value of $\varepsilon_{AB}$ leads not only to an increase in the aggregation number 〈A〉, but also to a weaker decrease of ${\langle A\rangle }_{L}$ with increasing distance *L* from the nanoparticle surface.

**Figure 11:**
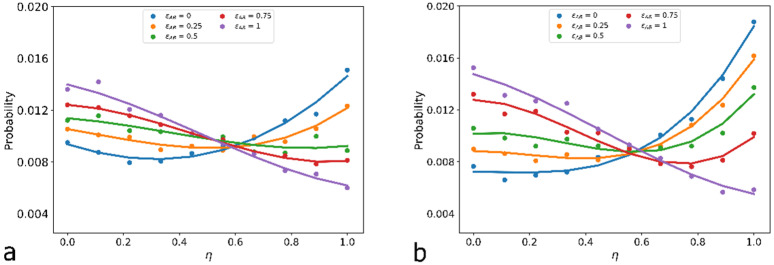
Distribution of A-B bonds over orientation η at $\varepsilon_{AA}=-8$ (**a**) and −10 (**b**) and different $\varepsilon_{AB}$ values.

Figure 11 presents the distribution of monomer units over their relative position η. In all the cases, the monomer units occupy a very arbitrary orientation: η varies over a wide range, and for each $\varepsilon_{AB}$ one can find a bond radius vector ${\vec{r}}_{AB}$, connecting A and B beads, both parallel to the radius vector ${\vec{r}}_{NA}$ and perpendicular to it. However, at $\varepsilon_{AB}$ = 0 the maximum of distribution is at η~1 and thus the most probable A-B bond orientation is *θ* = 0° or 180°. With $\varepsilon_{AB}$ = 1, the most probable state is η~0, which is perpendicular to the ${\vec{r}}_{NA}$ orientation of ${\vec{r}}_{AB}$: *θ* = 90°.

The value of $\varepsilon_{AB}$ with uniform over all η distributions was enacted to draw the conditional borderline between the compacted structured shell and multipetaler structure.

**Figure 12:**
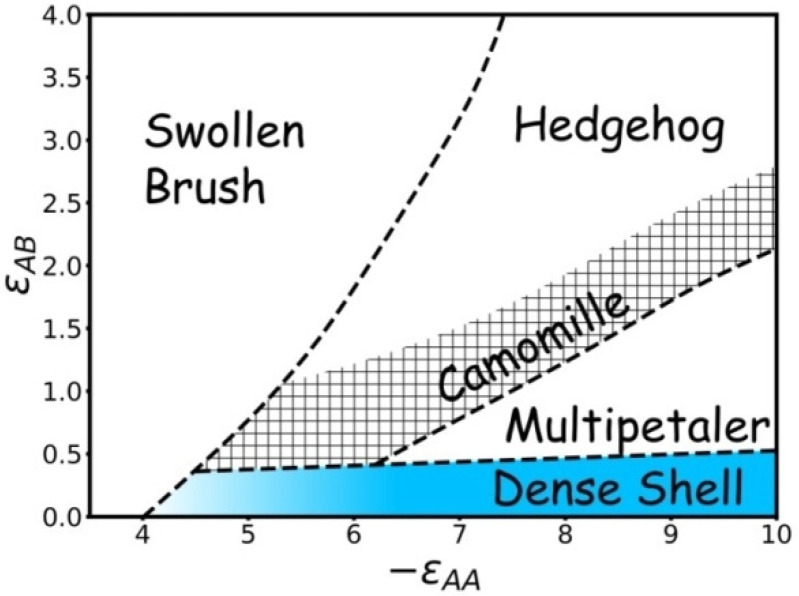
State diagram of the $\varepsilon_{AA\;}$vs. $\varepsilon_{AB}$ variables.

The results are summarized as a state diagram (Figure 12). The areas of the swollen brush, dense shell, hedgehog, chamomile, and multipetaler are highlighted. The stability regions of these structures depend on the quality $\varepsilon_{AA}$ of the solvent and the surface activity $\varepsilon_{AB}\;$of the monomer units. The stronger the A-B repulsion, the wider the field of swollen brush. At high $\varepsilon_{AB}$ values, the grafted layer either stays in a swollen state or adopts the hedgehog structure at low and high A-A attraction, respectively. With a small $\varepsilon_{AB}$, the swollen brush collapses smoothly to a dense shell along with $\varepsilon_{AA}$ growth. The multipetalers are observed when the A-A attraction is strong, and A-B repulsion is strong enough to destroy the dense shell but not too large to force macromolecules join into cylindrical spikes. The transformation of hedgehog to multipetaler proceeds through the chamomile structure where some spikes are connected into loops. The area where loops are observed is shaded.

## 4. Conclusions

In this paper, we performed computer modelling of a single spherical nanoparticle coated with an amphiphilic homopolymer and dissolved in a solvent that is selectively poor for main chains and good for pendant groups. It was found that the comb-like A-graft-B structure of monomeric units induces specific micro-segregation of incompatible A and B groups and leads to their self-assembly into the prolonged aggregates, vertical lamellae, and dense shells. The aggregates form a complex pattern on the nanoparticle surface. Most characteristics of the structures were distinguished, and their areas of stability were outlined in the state diagram. The distinguished structures are swollen brush; hedgehog, where the macromolecules join into the prolonged spike-like aggregates; chamomile, when the prolonged aggregates merge by their ends; multipetaler with the deflecting lamellae layers; and dense structured shells. The borderlines between structures were evaluated visually and through the calculations of aggregation numbers and parameters that are specially introduced in this paper. It was found that the aggregation number depends not only on the solvent quality for backbone A groups and on the degree of incompatibility between mainchain and pendant groups, but also on the distance from the nanoparticle surface. The gradual decrease in the aggregation number, due to a decrease in the effective grafting density, as one moves away from nanoparticle surface was first described in this paper and is called the branching effect. The branching effect makes the ending parts of aggregates more flexible and the sticking of their ends more profitable. As a result, the chamomile area, with aggregates forming loops, occupy a significant part of the structure diagram, whereas in the framework of analytical theory, where the branching effect was neglected [54], the chamomile area in the diagram is narrower. However, it is worthwhile to note that the relative position of the regions of various structures confirms the results of the previously published analytical theory.

It was found that the transition between structures proceeds smoothly and that the various elements, i.e., elongated aggregates and elongated aggregates with split and merging ends, can be implemented simultaneously on the surface of the nanoparticle. It is worthwhile to note that the described structures not only give different patterns on the surface, but also have a different number of unprotected hydrophobic groups and thus demonstrate significantly different behavior in solutions.

Polymer-coated nanoparticles, in which the sensitivity to external triggers is constructed by the inclusion of significantly different groups as pendants to mainchain, have become one of the main trends in research. In this regard, this paper reported the first detailed study of nanoparticles coated with A-graft-B macromolecules, which can serve as a guide for the targeted design of stimulus-sensitive nanoparticles and materials based on them.

## Data Availability

The data presented in this study are available on request from the corresponding authors.
